# Attitudes and perceptions of workplace intergenerational climate in Saudi universities: a cross-sectional study of nursing faculty and staff members

**DOI:** 10.3389/fmed.2025.1568196

**Published:** 2025-09-24

**Authors:** Regie B. Tumala, Abdualrahman S. Alshehry, Homood A. Alharbi, Isabelita N. Pandaan, Irene M. Roco, Ramon Perley M. Pandaan, Naif H. Alanazi, Abdulaziz M. Alotaibi, Mohammed A. Alfaifi

**Affiliations:** ^1^College of Nursing, King Saud University, Riyadh, Saudi Arabia; ^2^Nursing Department, College of Applied Medical Sciences, Shaqra University, Shaqra, Saudi Arabia

**Keywords:** nurse educator, nursing faculty, staff member, workplace intergenerational climate perspective, Saudi Arabia

## Abstract

**Background:**

In the first quarter of the 21st century, significant transformations have occurred within the workforce. One prominent change is the emergence of multigenerational workplaces, which now encompass four distinct generations. Given the scarcity of research focusing on the generational climate within nursing academia, this study seeks to examine the intergenerational workplace environment among nursing faculty and staff members. *Objective:* To assess nursing faculty and staff members’ attitudes and perceptions regarding colleagues of varying ages in their professional setting.

**Methods:**

This research employed a descriptive-correlational design. The current study employed the Workplace Intergenerational Climate Scale (WICS). Data collection was conducted from January 2024 to March 2024. All analyses were conducted utilizing SPSS version 28 statistical software.

**Findings:**

The current research indicated that nursing faculty and staff members held a modest perception of the intergenerational climate in their workplace, as evidenced by an overall mean score of 13.84 out of 20. Various demographic factors, such as ethnicity (nationality), educational level, and length of service, play a significant role in shaping these perceptions, thereby underscoring the intricate nature of intergenerational dynamics within nursing academia. This research is crucial for comprehending the dynamics of the work environment in nursing faculties across Saudi universities.

**Conclusion:**

The findings suggest that respondents held a moderately favorable view of their workplace’s intergenerational environment. By shedding light on the perceptions of intergenerational climate, it paves the way for improved collaboration, enhanced retention, and greater overall job satisfaction. The results indicate a promising intergenerational climate that fosters job satisfaction and diminishes stereotypes, which is vital for cultivating a more unified and effective educational atmosphere.

## 1 Introduction

In the first quarter of the 21st century, significant transformations have occurred within the workforce. One prominent change is the emergence of multigenerational workplaces, which now encompass four distinct generations (1). This four-generation workforce includes Baby Boomers (born 1946–1964), Generation X (born 1965–1980), Generation Y or Millennials (born 1981–1995), and Generation Z or Centennials (born 1996–2010s), all of whom are actively engaged in various professional environments (2, 3). Presently, the workforce is characterized by an unprecedented number of generational cohorts. Each generation brings unique personal, educational, and professional backgrounds, posing challenges for organizations (4). The increasing age diversity, with four generations collaborating in the workplace, is particularly noteworthy (5). Recent studies indicate that generational differences manifest in various aspects, including work values, communication styles, motivation, job satisfaction, work-life balance, and learning preferences (6). Consequently, effective leadership within organizations must adeptly manage these intergenerational dynamics (2). Furthermore, research has identified specific challenges in managing a multigenerational academic workforce, such as disparities in career expectations, skills, and experiences, as well as issues related to conflict resolution, strategic implementation, succession planning, and teamwork (7). Notably, demographic trends indicate that Generation Y (Gen Y) has emerged as the largest group entering the workforce in recent years (8).

In the global academic organization, various generational groups also exist as faculty and staff qualifications are required (9–11). In the Middle East for instance, to work in nursing higher education, a Bachelor of Science in Nursing (BSN) and relevant experience is needed (9). Higher positions require a Master’s or Doctorate (PhD) in nursing degree with specific clinical experience. This system is like the workforce in the nursing higher education in the Kingdom of Saudi Arabia (KSA) (12, 13). Specifically, a faculty member commonly holds a PhD while a staff member may have a BSN or MSN degree (12, 13). Adhering to the standardized qualification for entry level in the workforce in the academe can contribute to the generation gaps in the workforce which poses as a challenge (12, 13). In the Saudi Arabian context regarding the academic setting, gender segregation in public places, including state universities, constitutes the cornerstone of the interpretation of Islam in the KSA, in relation to having separate female and male campuses (14–16). Hence, the female and male campuses are separated in the two settings of the current study. This segregation presents several challenges within the KSA. For female students or researchers who are married, it is necessary for their husbands to accompany them during data collection on male campuses when recruiting male participants for research studies with self-administered surveys or face-to-face interviews. Despite indications of developments toward easing certain restrictions on Saudi women, they are still required to have a male guardian (a father or husband) who can make a range of significant decisions on their behalf. This development has resulted to extensive separate public spaces that are designated only for women (14–16). For males, they must obtain permission before accessing any female campus within the university (16).

Recent studies indicate that factors such as intergenerational inclusiveness, emotional connections, retention, and generational stereotypes are inversely associated with perceived age discrimination (17). Bae and Choi (18) argue that the relationship between chronological age and ageist attitudes in the workplace is not linear; notably, younger individuals often exhibit more ageist attitudes than their older colleagues. While there is existing research on intergenerational relationships across various work environments, investigations specifically targeting the educational sector, particularly higher education, remain limited (19). Furthermore, although there are a few studies addressing intergenerational dynamics within university settings, they primarily link these dynamics to issues of discrimination, job engagement, and professional satisfaction (19, 20), as well as to learning processes, knowledge sharing, and the mitigation of knowledge loss (19, 21). Given the scarcity of research focusing on the generational climate within nursing academia, this study aimed to investigate the attitudes and perceptions of nursing faculty and staff members regarding employees of varying ages within the workplace. Furthermore, it examined whether there are differences in the attitudes and perceptions of nursing faculty and staff members toward employees of different ages when categorized by gender, generation, nationality, language, education, work duration, job description, and university.

## 2 Materials and methods

This quantitative research employed a descriptive-correlational design. Data collection was conducted from January 2024 to March 2024. For the sample size, this study utilized the *A priori* computation software (G*Power version 3.1.9.7) with given values for effect size, alpha and power. When using the software, the analysis of variance (ANOVA) with four groups for work duration of the respondents had a medium effect size of 0.25, alpha error probability of 0.05 and power of 0.95 that yielded a minimum sample size of 400. The study utilized an online survey that involved the recruitment of a convenient sample of nursing faculty members, including teaching assistants, lecturers, assistant professors, associate professors, and full professors, along with staff members working as administrative personnel in the two study settings. Respondents were recruited through a convenience sampling method, if they worked, from the College of Nursing at University A in Riyadh and from the College of Applied Medical Sciences at University B in Shaqra, KSA. Respondents who were on study leave locally or abroad and on sabbatical leave were excluded from participating in the online survey.

The study employed the Workplace Intergenerational Climate Scale (WICS), a tool created by a multidisciplinary group of specialists in aging and workforce dynamics at the Mather LifeWays Institute on Aging, located in Evanston, Illinois, United States (3). The questionnaire is composed of two parts. In the first part of the tool, eight questions asked the demographics of the participants which include age, gender, generation, nationality, language, highest educational attainment, length of employment, and department assigned. The second part of the questionnaire consists of 20 items designed according to a 4-point Likert scale asking about workplace intergenerational climate. These questions are classified into five sections, including ‘lack of generational stereotypes (LGS)’ (four items), ‘positive intergenerational affect (PIA)’ (four items), ‘intergenerational contact (IC)’ (four items), ‘workplace generational inclusiveness’ (WGI)’ (four items), and ‘workplace intergenerational retention (WIR)’ (four items). In terms of the validity and reliability of applying the questionnaire in the current study, internal consistency values (using alpha Cronbach’s coefficient) were 0.65 (LGS), 0.68 (PIA), 0.62 (IC), 0.61 (WGI),0.60 (WIR), and 0.81 for the overall WICS, which was lower than (alpha = 0.87) King and Bryant’s (3) study, but still acceptable, exceeding the minimum recommendation of 0.80 (29) and also aligns with general guidelines established by Nunnally (30).

The researchers obtained ethical approval from the Research Center of the College of Nursing at University A and adhered to a process of informed consent for all respondents. It is important to highlight that all individuals approached for the study had the opportunity to voluntarily complete the questionnaire. Respondents were informed that they have the right to withdraw from the study at any point. They were assured that their responses are kept confidential and that their identities would not be disclosed in any research reports or publications. It was emphasized that respondents’ decision not to participate would not affect their employment status. Following the acquisition of ethical approval, the researchers requested support from the heads of the nursing departments to engage potential respondents from both the male and female campuses of the two study settings. The data collection process was expected to require approximately 10 to 15 min for each individual respondent to complete the online survey.

All analyses were conducted utilizing SPSS version 28 statistical software. Descriptive statistics were employed to evaluate the data pertaining to all study variables. Percentages were computed to ascertain the demographic characteristics of the respondents. Weighted means were calculated to assess the attitudes and perceptions of nursing faculty regarding workers of varying ages in the workplace. The independent samples t-test and ANOVA were utilized to compare the attitudes and perceptions of nursing faculty concerning workers of different ages. The tests of difference were categorized by gender with two groups, generation with two groups (Baby boomer had seven respondents, was not included in the test of difference analysis), nationality with two groups, language with three groups, education with three groups, work duration with four groups, job description with two groups, and university with two groups. The threshold for statistical significance was established at a probability value of 0.05.

## 3 Results

A total of 450 responses was retrieved from the online survey, with only 415 completed responses (more than the required minimum sample size of 400), which were subsequently included in the data analysis, yielding a response rate of 92.2%. As presented in Table 1, the ages of the participants varied from 20 to 77 years, with a mean age of 37.09 years (SD = 7.43). The demographic profile of the respondents indicated that a significant majority were female (65.3%), Saudi nationals (71.6%), millennials (64.3%), Arabic speakers (60.2%), and holders of baccalaureate degrees (54.2%). Furthermore, 60.7% of the respondents were employed as staff members, with 62.7% working at University A. Notably, nearly half of the participants (47.0%) reported having worked at the university for a duration of 6 to 10 years.

**Table 1:** Demographic characteristics of the respondents (*n* = 415).

Demographic Characteristics	Mean (SD)	Range
Age	37.09 (7.43)	20–77
	*f*	%
Gender		
Male	144	34.7
Female	271	65.3
Generation		
Baby boomer	7	1.7
Generation X	141	34.0
Generation Y/ Millennial	267	64.3
Nationality		
Saudi	297	71.6
Non-Saudi	118	28.4
Language		
English	75	18.1
Arabic	250	60.2
Both	90	21.7
Education		
Bachelor	225	54.2
Master	98	23.6
Doctorate	92	22.2
Work duration		
<1 year	28	6.7
1–5 years	92	22.2
6–10 years	195	47.0
>10 years	100	24.1
Job description		
Faculty member	163	39.3
Staff member	252	60.7
University		
Univeristy A	260	62.7
University B	155	37.3

f, frequency; %, percentage.

The average score for the Workplace Intergenerational Climate (WIC) was 13.84 (SD = 1.59), with a scoring range from 5 to 20. This suggests that respondents held a moderately favorable view of their workplace’s intergenerational environment. Among the various dimensions assessed, “Workplace General Inclusiveness” (WIG) garnered the highest positive evaluations, with a mean of 3.22 (SD = 0.50). This was followed by “Positive Intergenerational Affect” (PIA) at a mean of 2.90 (SD = 0.42), “Workplace Intergenerational Retention” (WIR) with a mean of 2.77 (SD = 0.66), and “Lack of Generational Stereotypes” (LGS) which had a mean of 2.57 (SD = 0.66). Conversely, “Intergenerational Contact” (IC) received the least favorable perceptions, reflected in a mean score of 2.38 (SD = 0.60). Detailed means for each subscale are provided in Table 2.

**Table 2:** Results of the descriptive analyses on the workplace intergenerational climate scale (*n* = 415).

Workplace intergenerational climate scale items	Mean	SD
Lack of generational stereotypes	2.57	0.66
1. Co-workers outside my generation are not interested in making friends outside their generation.^a^	2.61	0.86
2. Co-workers outside my generation complain more than co-workers my age do.^a^	2.60	0.86
3. Co-workers outside my generation usually talk about things that do not interest me.^a^	2.57	0.74
4. Co-workers outside my generation tend to work differently than co-workers my age do.^a^	2.48	0.83
Positive Intergenerational Affect	2.90	0.42
5. I feel comfortable when co-workers outside my generation try to make conversation with me.	2.99	0.62
6. I enjoy interacting with co-workers of different generations.	3.27	0.70
7. My co-workers outside my generation are interesting and unique individuals.	2.95	0.66
8. People work best when they work with others their same age.^a^	2.37	0.76
Workplace Generational Inclusiveness	3.22	0.50
9. I believe that my work environment is a healthy one for people of all ages.	3.17	0.69
10. Workers of all ages are respected in my workplace.	3.07	0.76
11. I am able to communicate effectively with workers of different generations.	3.34	0.60
12. Working with co-workers of different ages enhances the quality of my work life.	3.30	0.65
Workplace Intergenerational Retention	2.77	0.66
13. My co-workers make older workers feel they should retire.^a^	2.69	0.89
14. I feel pressure from younger workers to step down.^a^	2.80	0.90
15. I feel pressure from older workers to step down.^a^	2.81	0.86
16. In my workplace, qualified younger workers tend to be overlooked for promotions.^a^	2.78	0.69
Intergenerational Contact	2.38	0.60
17. How often do you have conversations with co-workers outside your generation?	2.68	0.74
18. How often do you have conversations with co-workers outside your generation relating to things other than work?	2.49	0.75
19. How often do you talk with co-workers outside your generation about your personal lives?	2.07	0.85
20. How often do you eat meals with co-workers outside your generation during the workday?	2.29	0.86
Overall score	13.84	1.59

^a^Reverse scored.

The findings from the analysis of the relationship between the demographic characteristics of respondents and their perceptions regarding various dimensions of WIC are presented in Table 3. The results indicated a weak negative correlation between age and WIR (*r* = −0.17, *p* = 0.001). Saudi nurses exhibited significantly higher scores in WIR (*t* = 7.76, *p* < 0.001) but lower scores in PIA (*t* = −1.98, *p* = 0.049) compared to their non-Saudi counterparts. Additionally, a significant variance in nurses’ perceptions of WIR was identified based on educational background (*F* = 17.33, *p* < 0.001) and length of service (*F* = 4.96, *p* = 0.002). Post-hoc analyses indicated that nurses holding a baccalaureate degree had more favorable perceptions of WIR than those with master’s (*p* = 0.003) and doctoral degrees (p < 0.001). Furthermore, nurses with 1–5 years of experience at the University reported more positive perceptions of WIR than those with 6–10 years of experience (*p* = 0.001). Staff members (*M* = 3.27, SD = 0.48) demonstrated more favorable perceptions of WGI compared to faculty members (*M* = 3.14, SD = 0.52, *t* = −2.64, *p* = 0.009). The nursing faculty and staff at University B expressed more positive views regarding their organization’s PIA (*t* = −3.57, *p* < 0.001) and IC (*t* = −2.46, *p* = 0.015), yet reported lower scores in WRI (*t* = 10.51, *p* < 0.001) than those at University A. No significant differences were found in perceptions of the various dimensions of WIC across different generational groups.

**Table 3:** Results of the tests of associations and differences on workplace intergenerational climate dimensions (*n* = 415).

Dimensions	LGS^a^			PIA^b^			WGI^c^			WIR^d^			IC^e^		
Demographic Characteristics	Mean (SD)	Test	*p*	Mean (SD)	Test	*p*	Mean (SD)	Test	*p*	Mean (SD)	Test	*p*	Mean (SD)	Test	*p*
Age		*r* = −0.04	0.434		*r* = 0.03	0.511		*r* = −0.07	0.158		*r* = −0.17	0.001**		*r* = −0.03	0.584
Gender															
Male	2.55 (0.63)	*t* = −0.36	0.721	2.86 (0.38)	*t* = −1.11	0.267	3.21 (0.49)	*t* = −0.36	0.717	2.69 (0.62)	*t* = −1.78	0.076	2.38 (0.49)	*t* = −0.09	0.926
Female	2.57 (0.67)			2.91 (0.43)			3.23 (0.50)			2.81 (0.67)			2.38 (0.65)		
Generation															
Generation X	2.48 (0.63)	*t* = −1.88	0.060	2.90 (0.38)	*t* = 0.07	0.943	3.25 (0.51)	*t* = 0.84	0.403	2.75 (0.68)	*t* = −0.51	0.611	2.38 (0.53)	*t* = −0.06	0.950
Generation Y/ Millennial	2.61 (0.67)			2.89 (0.43)			3.20 (0.49)			2.79 (0.65)			2.39 (0.63)		
Nationality															
Saudi	2.59 (0.68)	*t* = 1.21	0.228	2.87 (0.40)	*t* = −1.98	0.049*	3.23 (0.48)	*t* = 0.94	0.350	2.93 (0.58)	*t* = 7.76	<0.001***	2.39 (0.63)	*t* = 0.70	0.487
Non-Saudi	2.50 (0.62)			2.96 (0.45)			3.18 (0.53)			2.38 (0.68)			2.35 (0.51)		
Language															
English	2.72 (0.74)	*F =* 2.50	0.083	2.91 (0.41)	*F =* 0.38	0.683	3.18 (0.53)	*F =* 1.03	0.359	2.72 (0.72)	*F =* 1.00	0.369	2.48 (0.61)	*F =* 1.50	0.225
Arabic	2.52 (0.63)			2.88 (0.42)			3.21 (0.48)			2.81 (0.60)			2.35 (0.59)		
Both	2.56 (0.67)			2.93 (0.43)			3.28 (0.52)			2.71 (0.74)			2.41 (0.60)		
Education															
Bachelor	2.60 (0.70)	*F =* 0.75	0.474	2.87 (0.41)	*F =* 1.11	0.331	3.26 (0.48)	*F =* 2.43	0.089	2.93 (0.59)	*F =* 17.33	<0.001***	2.41 (0.63)	*F =* 1.16	0.316
Master	2.53 (0.63)			2.93 (0.42)			3.23 (0.52)			2.68 (0.70)			2.40 (0.62)		
Doctorate	2.51 (0.60)			2.93 (0.42)			3.12 (0.51)			2.49 (0.67)			2.30 (0.48)		
Work duration															
<1 year	2.49 (0.63)	*F =* 1.10	0.351	2.91 (0.45)	*F =* 0.58	0.626	3.16 (0.53)	*F =* 1.33	0.264	2.85 (0.55)	*F =* 4.96	0.002**	2.36 (0.49)	*F =* 0.30	0.827
1–5 years	2.62 (0.71)			2.90 (0.43)			3.15 (0.51)			2.96 (0.62)			2.40 (0.65)		
6–10 years	2.52 (0.62)			2.87 (0.41)			3.22 (0.49)			2.65 (0.67)			2.36 (0.61)		
>10 years	2.64 (0.69)			2.94 (0.41)			3.29 (0.47)			2.81 (0.65)			2.42 (0.56)		
Job description															
Faculty member	2.50 (0.63)	*t* = −1.65	0.100	2.90 (0.41)	*t* = 0.36	0.721	3.14 (0.52)	*t* = −2.64	0.009**	2.70 (0.61)	*t* = −1.80	0.073	2.35 (0.58)	*t* = −0.93	0.354
Staff member	2.61 (0.68)			2.89 (0.42)			3.27 (0.48)			2.82 (0.68)			2.40 (0.60)		
University															
University A	2.56 (0.66)	*t* = −0.07	0.941	2.84 (0.38)	*t* = −3.57	<0.001***	3.25 (0.50)	*t* = 1.73	0.085	3.02 (0.47)	*t* = 10.51	<0.001***	2.33 (0.56)	*t* = −2.46	0.015*
University B	2.57 (0.67)			2.99 (0.46)			3.17 (0.49)			2.35 (0.71)			2.48 (0.64)		

^a^Lack of Generational Stereotypes, ^b^Positive Intergenerational Affect, ^c^Workplace Generational Inclusiveness, ^d^Workplace Intergenerational Retention, ^e^Intergenerational Contact, SD, Standard deviation, r, Pearson *r* value; *t*, t-test value; F, ANOVA value. *Significant at 0.05 level, **Significant at 0.01 level, ***Significant at 0.001 level.

As presented in Table 4, there exists a weak negative correlation between age and the overall WIC (*r* = −0.11, *p* = 0.027). Saudi individuals reported more favorable perceptions of the WIC (M = 14.02, SD = 1.59) in comparison to their non-Saudi counterparts (*M* = 13.38, SD = 1.50, *t* = 3.78, *p* < 0.001). The one-way ANOVA indicated a statistically significant difference in overall WIC perceptions based on educational attainment (*F* = 6.88, *p* = 0.001), with individuals holding baccalaureate degrees exhibiting more positive views than those with doctorate degrees (*p* = 0.001). Additionally, staff members demonstrated superior overall perceptions of the WIC (*M* = 13.99, SD = 1.62) compared to faculty members (*M* = 13.59, SD = 1.50, *t* = −2.51, *p* = 0.012). Furthermore, employees at University A reported a more favorable perception of their university’s WIC (*M* = 14.00, SD = 1.55) than those at University B (*M* = 13.56, SD = 1.61, *t* = 2.79, *p* = 0.006). Lastly, no significant differences were found in overall WIC perceptions across different generational groups (*t* = −0.73, *p* = 0.464).

**Table 4:** Results of the tests of associations or differences on the overall workplace intergenerational climate (*n* = 415).

Demographic characteristics	Mean	SD	Statistical test	*p*
Age			*r* = −0.11	0.027*
Gender				
Male	13.69	1.46	*t* = −1.32	0.187
Female	13.91	1.65		
Generation				
Generation X	13.76	1.54	*t* = −0.73	0.464
Generation Y/ Millennial	13.88	1.61		
Nationality				
Saudi	14.02	1.59	*t* = 3.78	<0.001***
Non-Saudi	13.38	1.50		
Language				
English	14.00	1.71	*F* = 0.67	0.511
Arabic	13.77	1.54		
Both	13.89	1.62		
Education^a^				
Bachelor	14.06	1.61	*F* = 6.88	0.001**
Master	13.77	1.60		
Doctorate	13.35	1.41		
Work duration				
<1 year	13.77	1.43	*F* = 2.58	0.054
1–5 years	14.04	1.70		
6–10 years	13.62	1.54		
>10 years	14.09	1.58		
Job description				
Faculty member	13.59	1.50	*t* = −2.51	0.012*
Staff member	13.99	1.62		
University				
University A	14.00	1.55	*t* = 2.79	0.006**
University B	13.56	1.61		

^a^Bachelor versus Doctorate (*p* = 0.001). SD, Standard deviation; r, Pearson *r* value; *t*, *t*-test value; F, ANOVA value. *Significant at 0.05 level, **Significant at 0.01 level, ***Significant at 0.001 level.

## 4 Discussion

The current research indicated that nursing faculty and staff members held a modest perception of the intergenerational climate in their workplace, as evidenced by an overall mean score of 13.84 out of 20. Analysis of the association between the demographic characteristics of respondents and their perceptions of various aspects of the workplace intergenerational climate (WIC) demonstrated a weak negative correlation between age and workplace intergenerational relations (WIR). Comparable finding has been observed from a study conducted in South Korea which found that participants in the lowest quartile of the WIC, who experienced the highest levels of workplace ageism, scored significantly higher on both the Fraboni Ageism Scale (FAS) and the Workplace Sexism Culture Scale (WSCS) compared to those in the upper 75% (44.6 vs. 41.4 and 24.1 vs. 22.0, respectively) (22). Additionally, another South Korean study revealed that chronological age did not exhibit a linear correlation with ageist attitudes in the workplace; however, younger individuals tended to display more ageist sentiments than their older counterparts. Notably, participants in their thirties showed the greatest reluctance to collaborate with individuals from different generational backgrounds (18). Importantly, negative attitudes toward intergenerational collaboration in the workplace were found to be statistically significant in relation to ageist attitudes toward older adults in non-work settings (18). It is undeniable that each generation contributes uniquely and valuably which fosters a harmonious balance among generations is crucial for developing a diverse workforce that can effectively address various challenges in professional environments (23).

In terms of respondents’ nationality, Saudi nurses demonstrated markedly higher scores in Work Engagement Index (WIR) but significantly lower scores in Professional Identity Assessment (PIA) compared to their non-Saudi counterparts. Research conducted by Attar and Alsharqi (24) indicates that the workload of non-Saudi nurses is statistically significant in relation to nurse turnover. Furthermore, the correlation between nurse turnover and organizational culture is characterized by a strong and direct association. Factors affecting turnover among foreign (expatriate) nurses reveal that professional growth (mean score 4.1 ± 0.7) and development received the highest mean agreement scores (4.0 ± 1.1), while housing (2.3 ± 1.3) and hospital facilities (2.1 ± 1.0) received the lowest mean scores. The elevated turnover rates adversely affect organizational quality of care and the resources required for recruiting and training new personnel, prompting numerous researchers to explore potential causes and devise comprehensive strategies for staff retention (25).

A notable disparity in nurses’ perceptions of WIR was identified based on varying educational backgrounds and lengths of service. Post-hoc analyses indicated that nurses holding a BSN degree exhibited more favorable views regarding WIR compared to their counterparts with master’s and doctoral degrees. This finding is comparable with another study conducted in the KSA by Alzuman and Alzouman (26) which found that the educational attainment of Saudi nurses did not significantly influence their satisfaction levels concerning management and workplace conditions. Nonetheless, it is imperative to pursue advancements in nursing science to foster professional growth. Additionally, nurses with 1–5 years of experience at the University reported more positive perceptions of WIR than those with 6–10 years of experience. These findings contrast with research conducted in Egypt, which demonstrated no statistically significant differences in ethical work climate, workplace alienation, and the personal characteristics of study participants, concluding that individual traits do not affect the ethical work environment (27). To address the challenge of retaining seasoned nurses, proposals such as a reduced workweek and increased flexibility in scheduling have been suggested (26).

Staff members exhibited more favorable perceptions of WGI compared to faculty members. Comparably, a study conducted in Egypt by Gabra et al. (27) indicated that 68.4% of nurses reported experiencing a negative ethical work climate, while only 31.6% experienced a positive ethical work climate. This finding suggests a negative correlation between ethical work climate and workplace alienation. At University B, nursing faculty and staff held more optimistic views regarding their organization’s PIA, although their perceptions of WRI were less favorable than those of their counterparts at University A. No significant differences were found in the perceptions of various dimensions of WIC across different generations. Research conducted in the UK by Atay and Williams (1) highlighted that the unique socio-economic contexts of each generation influence their responses to broader employment trends and their relationship with work. Evidence concerning substantial generational differences in the workforce remains ambiguous and inconsistent, with a growing consensus suggesting that such differences are often exaggerated. Instead, attention should be directed toward the commonalities that connect various generations.

Finally, the relationship between age and overall WIC exhibited a weak negative correlation. This finding is supported by Sanches et al. (23) noting that all generational cohorts regard teamwork as essential for fostering conducive practice environments, albeit with varying levels of emphasis. Notably, younger generations are significantly more inclined to perceive work as a central aspect of their lives (1). Furthermore, Saudi individuals reported more favorable perceptions of the WIC compared to their non-Saudi counterparts. A one-way ANOVA indicated a statistically significant difference in overall WIC perceptions based on educational attainment, with individuals holding baccalaureate degrees demonstrating more positive views than those with doctorate degrees. Additionally, staff members exhibited more favorable perceptions of the WIC than faculty members. Duru and Hammoud (28) identified key strategies for senior leaders to enhance nurse retention, including effective communication, respect, competitive financial compensation, benefits, and appropriate recognition.

## 5 Conclusion

The investigation into the intergenerational climate perspectives among nursing faculty and staff members at Saudi universities revealed a nuanced understanding of intergenerational interactions. The findings showed that while there is an overall favorable (modest) view towards inclusivity and positive intergenerational relationships, concerns regarding intergenerational contact persist. Various demographic factors, such as age, ethnicity (nationality), educational level, length of service (work duration), job description and university where the respondents were affiliated, play a significant role in shaping these perceptions, thereby underscoring the intricate nature of intergenerational dynamics within nursing academia in the Saudi Arabian context. This research is crucial for comprehending the dynamics of the work environment in nursing faculties and staff members across Saudi universities. By shedding light on the perceptions of intergenerational climate, it paves the way for improved collaboration, enhanced retention, and greater overall job satisfaction. The results indicate a promising intergenerational climate that would foster enhanced job satisfaction and lessens stereotypes, which is vital for cultivating a more unified and effective educational atmosphere, particularly in nursing academia.

## 6 Implications and recommendations

Intergenerational collaboration is essential, as the current study findings highlighted the significance of fostering such collaboration within nursing teams (faculty and staff members) in the academia. Acknowledging and appreciating the varied experiences and viewpoints of different age groups (various generations) can significantly improve workplace morale. To facilitate mentorship, it is beneficial to develop programs that pair younger and older nursing faculty and staff members, thereby bridging generational disparity and promoting the exchange of knowledge, which ultimately enhances professional nursing practice in the academia both in theory and clinical training of nursing students.

Furthermore, nursing institutions should implement policies that advocate for inclusivity and respect across all generations, irrespective of age, gender, race (nationality), job description or social background, ensuring that every nursing faculty or staff member feels valued and motivated. It is also crucial to confront stereotypes; ongoing training and education aimed at challenging generational biases can enhance intergenerational interactions, thereby improving teamwork and collaboration. Finally, effective communication strategies tailored to the diverse needs of various age groups can foster understanding and cooperation, contributing to a more cohesive working environment in nursing academia.

By implementing the following recommendations, Saudi universities can cultivate a more favorable intergenerational environment that promotes collaboration and job satisfaction, that may enhance the quality of nursing education and produce competent nursing graduates. For example, fostering enhanced intergenerational contact: initiatives such as team-building activities or interdepartmental projects may be introduced to encourage interactions across generations (Baby boomers to Generation Y or Millennials), thereby improving the overall workplace atmosphere. Other recommendations include:

*Training and development*: It is essential to provide training programs that emphasize generational differences and inclusivity, which will help to foster understanding and cooperation among nursing faculty and staff members.
*Regular assessment*: Saudi nursing academic institutions should carry out periodic evaluations of the workplace climate to track shifts in attitudes and perceptions, and proactively address any emerging concerns in the future.
*Encourage participation and feedback*: Establishing platforms for nursing faculty and staff members to express their opinions and suggestions regarding workplace dynamics is crucial to ensure that all generations feel acknowledged and valued.
*Further research and improvement*: Ongoing research into intergenerational dynamics within nursing academia should be pursued on a broader scale such as including private nursing colleges and/or on a regional and national level to develop additional strategies and best practices, ensuring that they remain relevant in a changing workplace.

## Data Availability

The original contributions presented in the study are included in the article/supplementary material, further inquiries can be directed to the corresponding author/s.
